# A novel benzothiazole derivative induces apoptosis via the mitochondrial intrinsic pathway producing antitumor activity in colorectal cancer

**DOI:** 10.3389/fphar.2023.1196158

**Published:** 2023-05-23

**Authors:** Jing Zhou, Rongce Zhao, Haoxuan Zhou, Shuping Yang, Feiyan Tao, Yongmei Xie, Hongli Wang, Jingping Yun

**Affiliations:** ^1^ Collaborative Innovation Center for Cancer Medicine, State Key Laboratory of Oncology in South China, Department of Pathology, Sun Yat-sen University Cancer Center, Guangzhou, China; ^2^ Collaborative Innovation Center for Cancer Medicine, State Key Laboratory of Oncology in South China, Department of Liver Surgery, Sun Yat-sen University Cancer Center, Guang Zhou, China; ^3^ State Key Laboratory of Biotherapy and Cancer Center, West China Hospital, Sichuan University, Chengdu, China; ^4^ Department of Pharmacy, The Third Affiliated Hospital (The Affiliated Luohu Hospital) of Shenzhen University, Shenzhen, China; ^5^ Research and Development Centre, China Tobacco Sichuan Industrial Co., Ltd., Chengdu, China; ^6^ School of Pharmacy, Chengdu Medical College, Chengdu, China

**Keywords:** colorectal cancer, benzothiazole derivative, apoptosis, small molecular drug, mitochondrial intrinsic pathway

## Abstract

**Background:** Colorectal cancer (CRC) is one of the most common malignancies causing the third highest mortality rate in the world. It is particularly urgent to explore effective therapeutic strategies to overcome this disease. We identified a novel benzothiazole derivative (BTD) that may serve as a potentially effective agent against CRC.

**Method:** MTT assays, cell colony formation assays, EdU staining assays, flow cytometry, RNA-seq, Western blotting, and migration and invasion assays were used to examine the effects of BTD on cell proliferation, apoptosis, metastasis, and the cell cycle. The antitumor activity of BTD *in vivo* was investigated in a CT26 tumor-bearing mouse model. Immunohistochemistry (IHC) was performed to examine the protein expression in mouse tumors. Hematology, biochemical analysis, and H&E staining were used to assess the biosafety of BTD.

**Results:** We observed that BTD suppressed cell proliferation and metastasis and promoted the apoptosis of tumor cells *in vitro*. Treatment with BTD at a tolerable dose significantly reduced tumor growth in CT26-tumor-bearing mice and appeared to be safe. Treatment of BTD induced apoptosis by increasing the generation of reactive oxygen species (ROS) and evoking the loss of mitochondrial transmembrane potential. Overall, BTD suppressed cell proliferation and metastasis, and induced apoptosis of colorectal tumor cells through the ROS–mitochondria-mediated apoptotic pathway. The preliminary proof of the antitumor activity and relative safety of BTD were validated in a mouse model.

**Conclusion:** Our findings suggest that BTD could serve as a potentially safe and effective candidate for CRC treatment.

## Introduction

CRC is one of the most common malignant tumors. Its incidence ranks third among all malignant tumors, and mortality caused by it ranks second (Sung et al., 2021). Since CRC is usually asymptomatic in the early stage, many patients are diagnosed at an advanced stage, lose the opportunity of surgery, and can only receive drug therapy such as chemotherapy and targeted therapy (Simard et al., 2019; Biller and Schrag, 2021). Unfortunately, a significant proportion of patients were resistant to chemotherapy or targeted therapy, owing to tumor heterogeneity and individual differences (Kuipers et al., 2015; Bien and Lin, 2021). Hence, it seems particularly urgent to develop new small molecule anti-CRC agent.

Benzothiazole consists of a heterocyclic ring system fused with a thiazole moiety and exerts a wide spectrum of biological effects, including antitumor, antimicrobial, antioxidant, and anti-inflammatory activities (Irfan et al., 2020; Zhilitskaya et al., 2021). Thus, the potential pharmacological applications of a range of benzothiazole derivatives aroused great interest of researchers (Wang et al., 2011). In the last decade, many studies have evaluated the antitumor activities of novel benzothiazole derivatives (Ahmed et al., 2012; Kamal et al., 2015; Saifutiarova et al., 2021). Mohsen et al. found that two new benzoxazole derivatives, compounds 7a and 8e, were promising antitumor agents for breast cancer (Omar et al., 2021). In recent years, our team has been focusing on the synthesis of novel benzothiazole derivatives and the evaluation of their biological activity and antitumor potential.

To promote apoptosis is one of the promising targets of antitumor drug research. In general, antitumor agents induce apoptosis in tumor cells mainly through two pathways: the mitochondria-mediated intrinsic pathway and the cell death receptor-mediated extrinsic pathway. Bcl-2 family members are critical regulators of the intrinsic pathway of apoptosis. Mitochondrial integrity is compromised when anti-apoptotic proteins of this family are inhibited and/or pro-apoptotic members are activated, giving rise to the release of cytochrome C, which interacts with ATP and Apaf-1, and then binds to pro-caspase-9. Cleaved caspase-9 sequentially activates the downstream effector proteins caspase-3 and/or caspase-7. On the other hand, in the extrinsic pathway, the ligation of death receptors leads to activation of the protease caspase-8, which activates downstream effector caspase-3 and/or caspase-7, thereby inducing cell apoptosis. Finally, both the intrinsic and extrinsic pathways result in DNA degradation, chromatin condensation, cell shrinkage, and the formation of apoptotic bodies (Fesik, 2005; Bedoui et al., 2020; Carneiro and El-Deiry, 2020). In this study, we demonstrated that a novel BTD, *N*-2-Benzothiazolyl-4-chloro-2-pyridinecarboxamide, could induce apoptosis in CRC cells via the mitochondrial apoptotic pathway and decrease cell invasion and migration, to inhibit tumor growth.

## Methods and materials

### Reagents

A novel BTD (chemical formula C_13_H_8_CIN_3_OS, *N*-2-Benzothiazolyl-4-chloro-2-pyridinecarboxamide) was synthesized by our team. The structure of BTD was confirmed using hydrogen-1 nuclear magnetic resonance (^1^H-NMR) and mass spectrometry. BTD was prepared as a 20 mM stock solution in dimethyl sulfoxide (DMSO) and stored at −80°C. For *in vitro* assays, the stock solution was diluted to an appropriate concentration in relevant assay mediums, and 0.1% DMSO was used as a vehicle control. For *in vivo* experiments, BTD was dissolved in 25% (v/v) aqueous Cremophor EL/ethanol (50:50, Sigma Cremophor EL, 100% ethyl alcohol) and administered at 10–20 mg/kg of body weight. 3-(4, 5-dimethylthiazol-2-yl)-2, 5- diphenyltetrazoliumbromide (MTT), DMSO, Rh123, and 2′,7′-dichlorodihydrofluorescein diacetate (DCFH-DA) were purchased from Sigma-Aldrich (St. Louis, MO, United States). The primary antibodies were purchased from Cell Signaling Technology (Beverly, MA, United States), Abcam (Cambridge, United Kingdom) and Santa Cruz (Dallas, Texas, United States). Apoptosis detection kits (annexin V–fluorescein isothiocyanate) and EdU staining kits were purchased from Beyotime Biotechnology (Shanghai, China).

### Cell lines and cell culture

The human colorectal cell lines HCT116, HT29, SW48, SW480, SW620, and DLD-1, and the mouse colorectal cell line CT26 were purchased from the American Type Culture Collection (ATCC, Manassas, VA, United States). All cell lines were authenticated in this study before use (GeneCreate Biotech). The cells were cultured in Roswell Park Memorial Institute-1640 (RPMI-1640) medium or Dulbecco’s modified Eagle’s medium (DMEM) supplemented with 10% heat-inactivated fetal bovine serum (FBS, Thermo-Fisher Scientific HyClone, United States) at 37°C under 5% CO_2_.

### Cell viability assays

The cytotoxic activity of BTD was evaluated using MTT assays. Cells (4 × 10^3^ cells/well) were plated in a 96-well plate and incubated overnight. Then the cells were treated with BTD (2.5, 5, or 10 μM) or vehicle for 24–72 h. MTT solution (10 µL/well) was then added and the cells were incubated for about 2–4 h at 37°C.The precipitated formazan crystal was redissolved in 150 mL of DMSO. The absorbance was measured using a paradigm molecular device at 450 nm (Tecan Infinite 200 Pro).

### Cell colony formation assays

Briefly, cells (400–600 cells/well) were seeded in a six-well plate. After incubation for 24 h, cells were treated with the indicated concentration of BTD for 12 more days. Subsequently, the cells were fixed with methanol for 15 min, stained with 0.1% crystal violet for another 15 min, and then counted under an optical microscope (>50 cells).

### EdU staining assays

For EdU staining assays, cells (1 × 10^4^ per well) were seeded in a 96-well plate and treated with the stated concentration of BTD for 24 h. The cells were first fixed in methanol for not less than 15 min, and then permeated using 0.1% Triton X-100. After blocking with 3% bovine serum albumin for 1 h, the cells were treated with 10 µL of EdU solution for another 18 h. After Hoechst staining (Dojindo, Kumamoto, Japan), the cells were observed and photographed with a fluorescence microscope.

### RNA sequencing

RNA sequencing was performed in HCT116 cells treated with BTD for 24 h. Total RNA was extracted and sequenced by The Beijing Genomics Institute (Shenzhen, China). Gene expression values were represented by expectation maximization data normalized within each sample to the upper quartile of the total reads, and high-quality clean data is the basis of all analysis in this study. The biological functions and signal pathways of BTD were enriched by gene set enrichment analysis (GSEA) and KEGG subsets analysis, which are shown as normalized enrichment scores. These results accounting for the size and degree to which a gene set is overrepresented at the top or bottom of the ranked list of genes (nominal *p*-value < 0.05 and FDR ≤ 0.25).

### Apoptosis detection assays

Cell apoptosis was measured using Apoptosis Detection Kits. Briefly, 1 × 10^5^ cells per well were seeded in a six-well plate, incubated overnight, and then treated with BTD in specified concentrations for the indicated time. The cells were then harvested, washed twice with cold phosphate-buffered saline and then stained with annexin V/propidium iodide (PI). The levels of apoptosis were detected using a flow cytometer (FCM).

### Western blotting

Cells were lysed in RIPA buffer and then centrifuged at 12,000 rpm for 15 min at 4°C to clarified the protein supernatant. The protein concentrations were measured using BCA assay kits (Thermo, Waltham, United States). The same amount of protein from each sample was loaded into each well and separated using electrophoresis (Bio-Rad, Hercules, United States) in a 10% sodium dodecyl sulfate-polyacrylamide gel (SDS-PAGE). Proteins were transferred to polyvinylidene difluoride (PVDF) membrane (Millipore, Billerica, United States) by electrophoresis, then blocked with 5% BSA at 25°C for 1 h. Subsequently, the PVDF membrane was subjected to primary antibodies at 4°C overnight, then washed in 0.1% TBS/T buffer at least three times, and incubated with secondary antibodies at 25°C for 1 h. The reactive bands were identified using enhanced chemiluminescence kits (Bio-Rad). The following antibodies were used at 1:1,000 for immunoblotting: mouse anti-Bcl-2 (cat. No. 15071S), rabbit anti-Bax (cat. No. 41162), rabbit anti-Bim (cat. No. 2933S), rabbit anti-Bad (cat. No. 9268S), rabbit anti-caspase-3 (cat. No. 9662S), rabbit anti-caspase-9 (cat. No. 9502S), rabbit anti-TIMP2 (cat. No. 5738), rabbit anti-Cyto C (cat. No. 11940), and rabbit anti-COX IV (cat. no. 4850) from Cell Signaling Technology; mouse anti-Bcl-x (cat. no. SC-56021, Santa Cruz Biotechnology); rabbit anti-MMP1 (cat. no. ab52631, abcam); rabbit anti-MMP9 (cat. no. AF0220, affbiotech); mouse anti-β-actin (cat. no. AF0003, Beyotime).

### Mitochondrial membrane potential and ROS detection assays

Mitochondrial Membrane Potential was detected by FCM with Rh123 staining. Briefly, the cells were seeded in six-well plates, and then treated with indicated concentrations of BTD for 24 h. The cells were then incubated with DCFH-DA solution or Rh123 solution and the changes in ROS or ΔΨm were measured using FCM.

### Migration and invasion assays

To measure cell migration, a total of 5 × 10^4^ cells were resuspended in 100 μL serum-free medium were added to the upper chamber (8 μM pore size, Millipore), and 600 μL of medium with 10% FBS was filled into the bottom. Different concentrations of BTD (0, 2.5, 5, and 10 μM) were added into both chambers. Cells were allowed to migrate through the pores for about 48 h, after which the cells remaining in the upper chamber were discarded. For invasion assays, the upper surface of chambers was covered with diluted Matrigel (BD Biosciences, United States). Samples of 5 × 10^4^ cells suspended in 100 μL of serum-free medium were added into the upper layer of the chamber. After that the cells were treated with a specific concentration of BTD. The bottom chambers were added with 600 μL of medium containing 10% FBS. After incubation for 48 h, non-migrated cells remaining on the upper surface of the filter were discarded, and the lower surface counterpart was fixed with 4% paraformaldehyde and stained with 0.5% crystal violet. Then the migrating and invading cells were photographed under a light microscope.

### Wound healing assays

Wound healing assays were used to assess cell migration. When the tumor cells grew to approximately 80% abundance, the cell monolayer was scratched with a sterile 10-µL pipette tip. After treatment with different concentrations of BTD for 48 h, the cells were fixed with methanol and photographed. The inhibition rate was expressed as the percentage of migrated cells, compared with the untreated group.

### Cell cycle detection assays

The cell cycle distribution was determined by FCM. Tumor cells were firstly treated with various concentrations of BTD for 24 h, and then were washed twice with cold PBS. Subsequently, tumor cells were fixed with 75% ethanol overnight. Finally, tumor cells were incubated with 500 μL of hypotonic solution consisting of 50 μg/mL PI, 0.1% Triton X-100, and 0.1% sodium citrate, in the dark for 30 min. The data was analyzed using the Flow Jo software.

### Animal experiments and evaluation of toxicity

All animal experiments were performed in accordance with disciplines of the Institutional Animal Care and Treatment Committee of Sichuan University (Permit Number: 20200323-3). Female BALB/c mice aged 6 to 8 weeks (HFK Biosciences Co., Ltd., Beijing, China) were housed in a specific-pathogen-free (SPF) condition facility. CT26 cells (0.5 × 10^6^) were inoculated subcutaneously in the right flank of mice. Tumor-bearing mice were randomly divided into three groups, when visible tumors reached about 100 mm^3^. One group received an intraperitoneal injection of 10 mg/kg BTD, one received 20 mg/kg BTD, and one received vehicle, once daily for 24 consecutive days. Tumor volumes were calculated as follows: Tumor volume (mm^3^) = 0.52× length × width^2^. Tumor inhibition rates (TIs) were calculated by the formula: TI = (V_control_–V_experiment_)/V_control_ × 100%. All mice were killed by cervical dislocation at the end of experiments. To identify any toxicity produced by BTD, body weight, anorexia, diarrhea, and other clinical symptoms were continuously monitored during the experiment. Hematoxylin-eosin (H&E) staining was performed on major organs (heart, liver, spleen, lung, and kidney) for histopathologic examination, and blood routine and biochemical analyses were performed as well.

### IHC

IHC staining of tumor section was adopted to detect changes in important molecules. Cleaved caspase-3, MMP9, and Ki-67 were examined to determine the effect of BTD on cell proliferation and apoptosis *in vivo*. Representative images were captured with an optical microscope.

### Statistical analyses

Data are presented as the mean ± standard deviation. Paired Student’s *t*-tests, Pearson’s χ2 tests, and two-way ANOVA tests were used to test the differences between groups. Results for which *p* < 0.05 (two-tailed) were considered to be statistically significant (*p < 0.05, **p < 0.01, and ***p < 0.001).

## Results

### Chemical information about BTD

**FIGURE 1:**
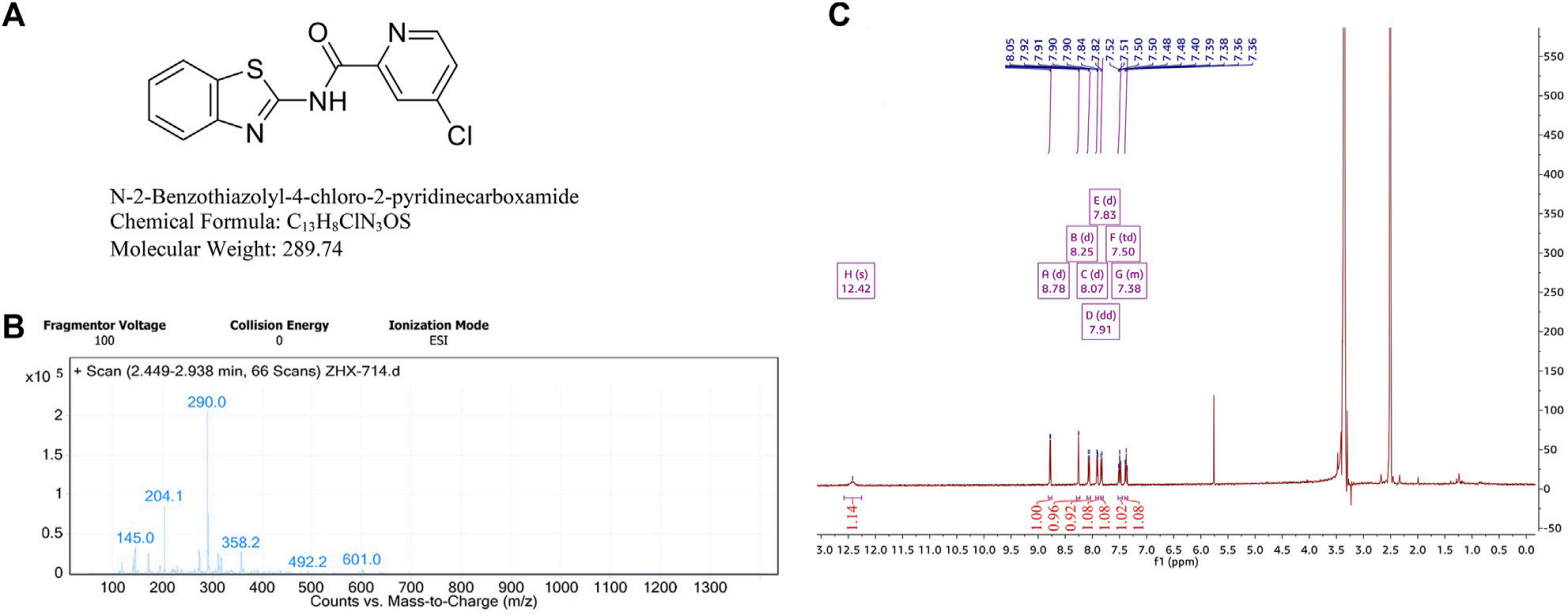
Chemical information about BTD. **(A)** The chemical structure and formula of BTD. **(B)** High resolution mass spectrometry of BTD, (*m/z* + H)^+^ = 290.0. **(C)** ^1^H-NMR spectra of BTD.

Our group has synthesized a novel BTD, *N*-2-Benzothiazolyl-4-chloro-2-pyridinecarboxamide. The chemical structure of BTD (C_13_H_8_CIN_3_OS) is shown in Figure 1A. The molecular weight is 289.74. Chemical information about BTD was obtained using mass spectrometry and ^1^H-NMR (Figures 1B, C).

### BTD suppressed cell proliferation

**FIGURE 2:**
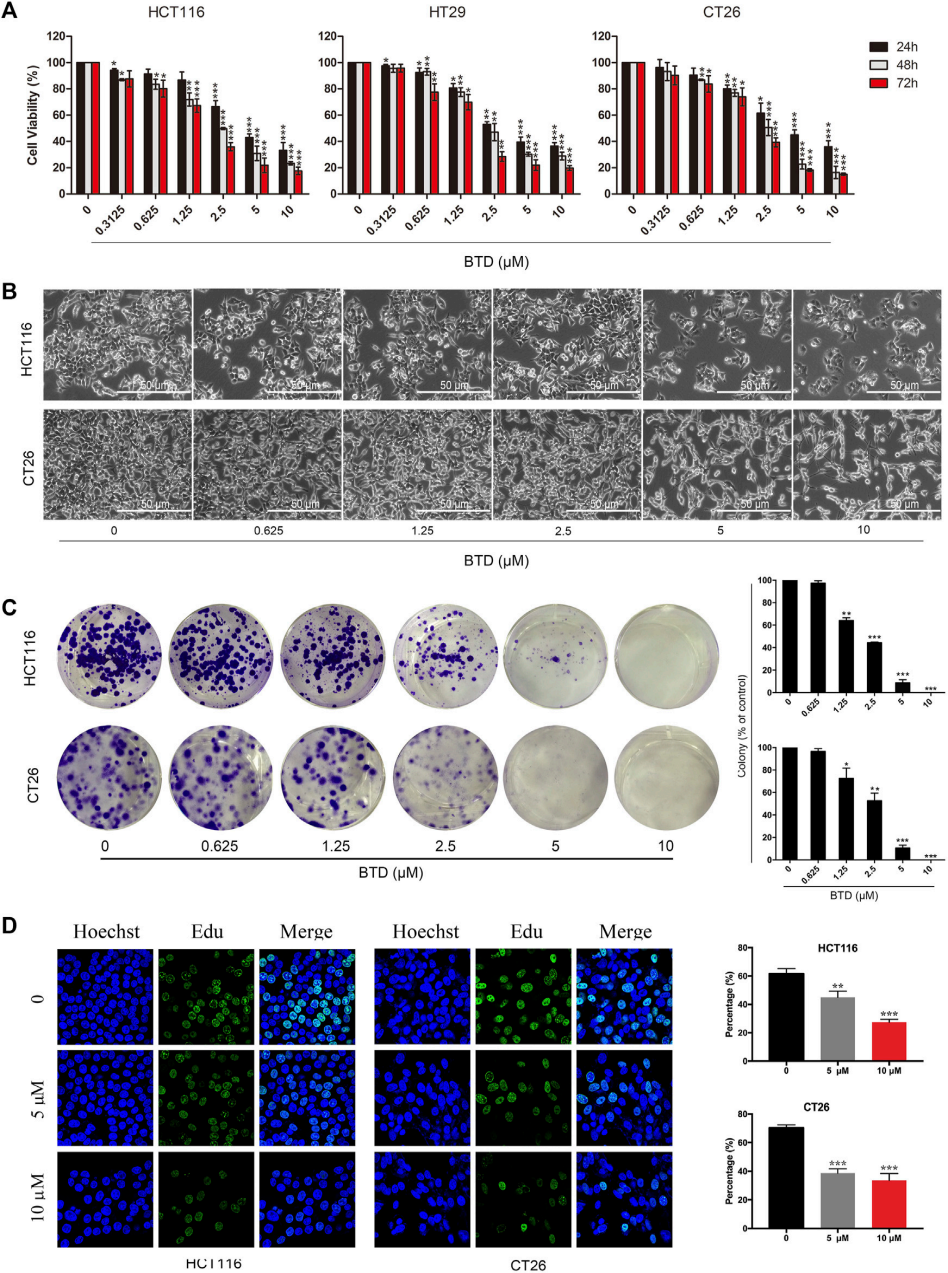
BTD suppressed cell proliferation. **(A)** Proliferation of HCT116, HT29, and CT26 cells was evaluated using MTT assays. Cells were treated with BTD (2.5, 5, or 10 μM) or vehicle for 24–72 h. **(B)** Cell morphology changes were observed by optical microscopy (×200). Cells were treated with a gradient concentration of BTD (0, 0.3125, 0.625, 1.25, 2.5, 5, and 10 μM) for 24 h. **(C)** Colony formation of HCT116 and CT26 cells after treatment with BTD. The results are presented as surviving colonies. **(D)** EdU staining assays showed the replication of DNA in HCT116 and CT26 cells treated with BTD (5 or 10 μM) or vehicle. Blue staining indicates the cell nucleus, and green staining indicates duplicated cells.

The effect of BTD on the viability of CRC cells was examined using MTT assays. Table 1 shows that BTD reduced the viability of tumor cells, with an IC_50_ of about 7.5 μM after treatment for 48 h. As shown in Figure 2A, treatment with 2.5, 5, and 10 μM BTD inhibited cell proliferation in a time- and concentration-dependent manner. After treatment with BTD, the CRC cells were observed to assume a circular morphology. As the BTD concentration was increased, the cells began to shrink, rotate, loosely arranged, weakly adhered, and even float in the nutrient medium (Figure 2B). To further determine whether BTD could inhibit the proliferation of CRC cells, colony formation assays were performed. BTD was found to attenuate the colony formation ability of CRC cells in a concentration-dependent manner (Figure 2C. As shown in Figure 2D, EdU-positive cells were noticeably reduced after treatment with BTD, as detected using EdU staining assays. Collectively, these findings indicated that BTD suppressed the proliferation of CRC cells.

**TABLE 1:** Effects of BTD on the viability of CRC cell lines.

Cell lines	Cells type	IC_50_ (μM)
HCT116	Human colorectal carcinoma cell line	6.48
HT29	Human colorectal adenocarcinoma cell line	7.17
SW48	Human colorectal adenocarcinoma cell line	8.79
SW480	Human colorectal adenocarcinoma cell line	8.68
DLD-1	Human colorectal adenocarcinoma cell line	7.92
SW620	Human colorectal adenocarcinoma cell line	8.15
CT26	Mouse colon carcinoma cell line	5.62

The CRC, cells were treated with a gradient concentration of BTD, for 48 h. IC_50_ was detected using MTT, assays. Data are from three parallel experiments.

### BTD induced apoptosis

**FIGURE 3:**
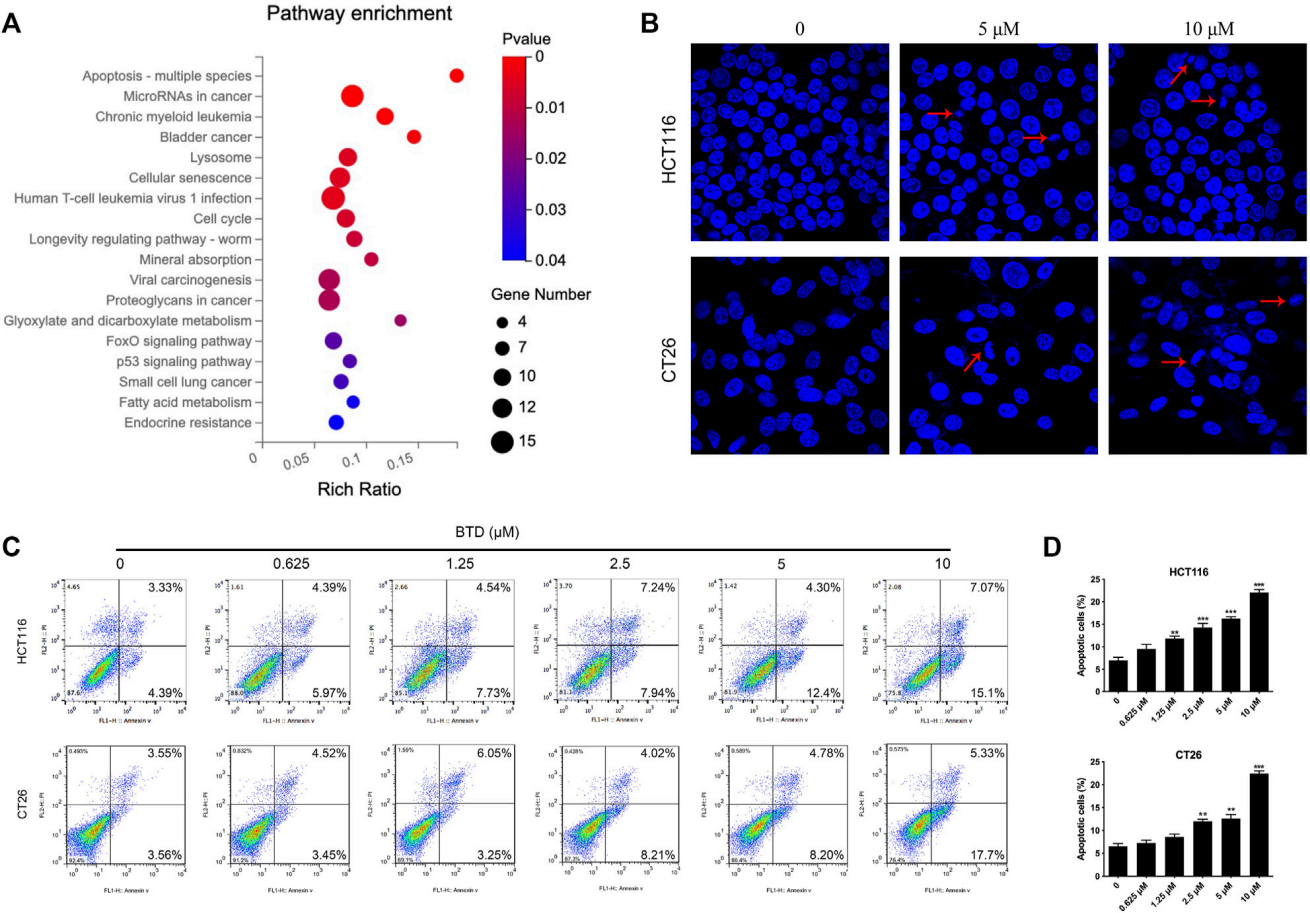
BTD induced apoptosis. **(A)** KEGG gene enrichment analysis of HCT116 cells treated with 5 μM BTD for 24 h. **(B)** Cell nuclear changes were detected by Hoechst 33,258 staining after treatment with BTD (0, 5 or 10 μM) for 24 h, then visualized using a fluorescence microscope (×200). **(C)** Cells were treated with BTD (0, 0.625, 1.25, 2.5, 5, or 10 μM) for 24 h, then apoptosis was evaluated using flow cytometry. **(D)** Levels of apoptosis. Bars represent mean ± standard deviation.

To reveal the mechanism underlying the suppressing of cell proliferation by BTD, transcriptional profiling of HCT116 cells was performed using RNA sequencing. Subsequent KEGG gene enrichment analysis indicated that differentially expressed genes (DEGs) were predominantly enriched in the apoptosis pathway (Figure 3A). Hoechst results showed nuclear fragmentation, nuclear condensation, bright blue fluorescence was observed after BTD treatment, indicating that apoptosis was induced in CRC cells (Figure 3B). FCM assays demonstrated a significant increase in the proportion of apoptotic cells after BTD treatment, compared with the control group (Figures 3C, D), suggesting that BTD promoted the apoptosis of CRC cells.

### BTD induced apoptosis via the mitochondrial apoptotic pathway

**FIGURE 4:**
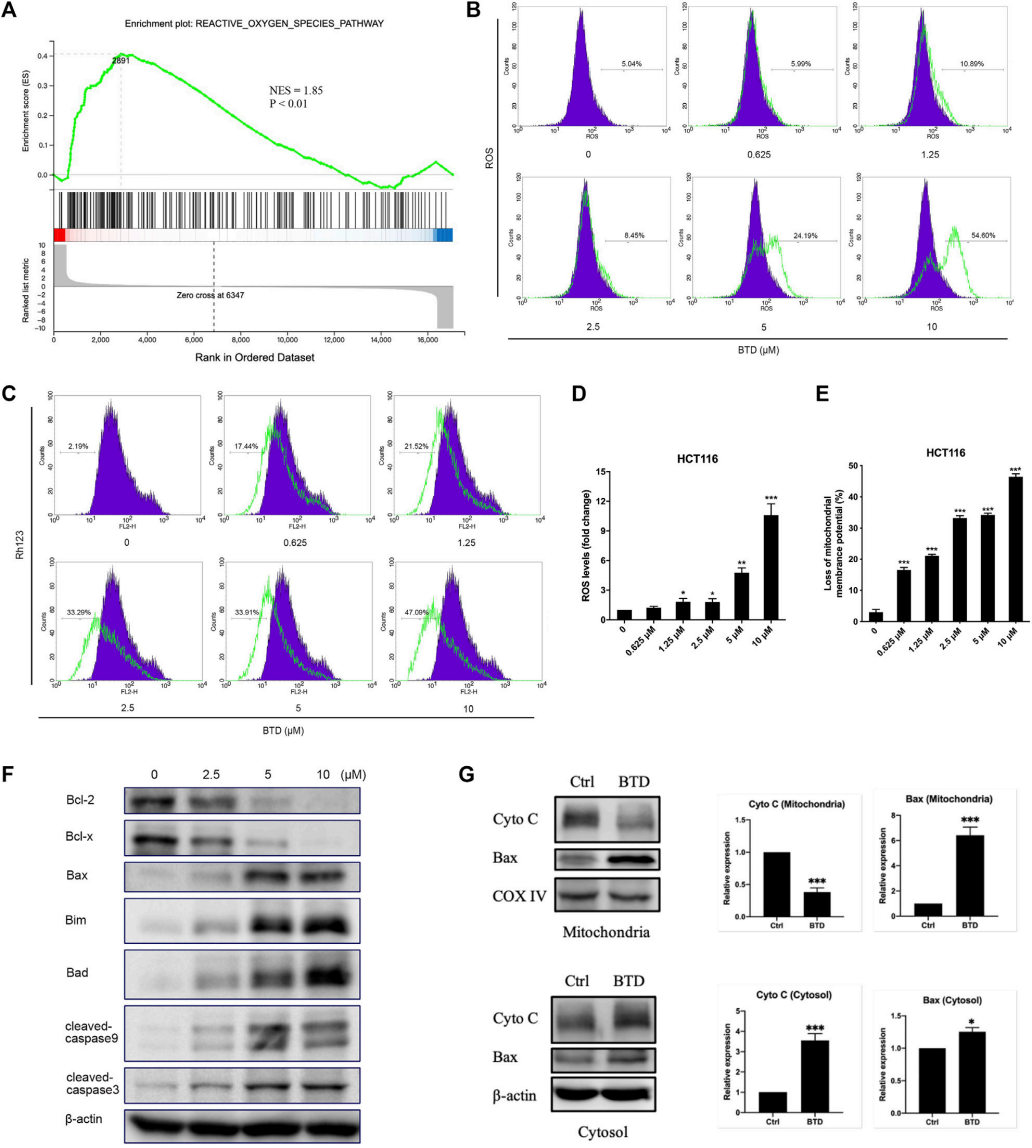
BTD induced apoptosis via the mitochondrial apoptotic pathway. **(A)** GSEA analysis of HCT116 cells treated with 5 μM BTD for 24 h **(B,C)** Cells were treated with BTD (0, 0.625, 1.25, 2.5, 5, or 10 μM) for 24 h and stained with 10 μM DCFH-DA solution to detect ROS levels **(B)**, or stained with Rh123 to detect changes in ΔΨm **(C)**, then measured using flow cytometry. **(D,E)** ROS and ΔΨm levels. Bars represent mean ± standard deviation. **(F)** HCT116 cells were treated with 5 μM BTD for 24 h, then the expression of the anti-apoptotic proteins Bcl-2 and Bcl-x, the pro-apoptotic proteins Bax, Bim, and Bad, the caspase family proteins cleaved caspase-9 and caspase-3 were detected using Western blotting. **(G)** HCT116 cells were treated with 5 μM BTD for 24 h, and the expression of Cyto C and Bax in the mitochondria and cytoplasm were detected using Western blotting.

We next explored the way in which BTD induced apoptosis. There are two main pathways of apoptosis: the mitochondrial-mediated apoptosis, and the death receptor pathway. Mitochondria are the main sites of ROS production in cells. The accumulation of excessive ROS disrupts the integrity of the mitochondrial membrane, causes the opening of mitochondrial membrane transport pores, reduces ΔΨm, and thus leading to mitochondrial pathway-mediated apoptosis (Zorov et al., 2014; Srinivas et al., 2019). The GSEA results indicated that BTD promoted the activation of the ROS pathway (Figure 4A), which is an integral part of the mitochondrial apoptotic pathway. The amount of ROS accumulated in CRC cells was detected by FCM analysis after staining with DCDH-DA reagent. ROS was observed to increase in a concentration-dependent manner after BTD treatment (Figures 4B, D). Fluorochrome Rh123, a mitochondria-specific, voltage-dependent dye solution, was used to detect changes of ΔΨm in tumor cells. ΔΨm was significantly decreased in tumor cells after BTD treatment, suggesting that the apoptosis induced by BTD may be mediated by the mitochondrial pathway (Figures 4C, E).

Bcl-2 family members are the key regulators of mitochondria-mediated apoptotic pathway. In this family, Bcl-2 and Bcl-x act as anti-apoptotic roles, whereas Bax, Bim, and Bad are pro-apoptotic proteins. The critical downstream proteins are cleaved during apoptosis (Letai, 2008; Singh et al., 2019). To determine whether BTD-induced apoptosis in CRC cells was through the mitochondria-mediated apoptotic pathway, Western blotting was performed. The results showed that the expression of Bax, Bad, and cleaved caspase-3 were upregulated, whereas the expression of Bcl-2 and Bcl-x were downregulated after BTD treatment (Figure 4F).

Once the pro-apoptotic proteins of Bcl-2 family are activated and/or anti-apoptotic counterparts are inhibited, mitochondrial integrity will be disrupted, followed by cytochrome C release (Bedoui et al., 2020). We also detected changes in the levels of Cyto C and Bax in the mitochondria and cytoplasm. Mitochondrial and cytoplasmic protein samples were prepared using mitochondrial extraction kits, and the protein levels were assessed using Western blotting. After treatment with BTD, the expression of Cyto C decreased in the mitochondria and increased in the cytoplasm, whereas the expression of Bax significantly increased in the mitochondria and slightly increased in the cytoplasm (Figure 4G), suggesting that BTD could induce translocation of Bax from the cytoplasm to the mitochondria and promote release of Cyto C from the mitochondria to the cytoplasm. These data supported that BTD induced apoptosis through the mitochondrial apoptotic pathway.

### BTD inhibited the migration and invasion ability of cells

**FIGURE 5:**
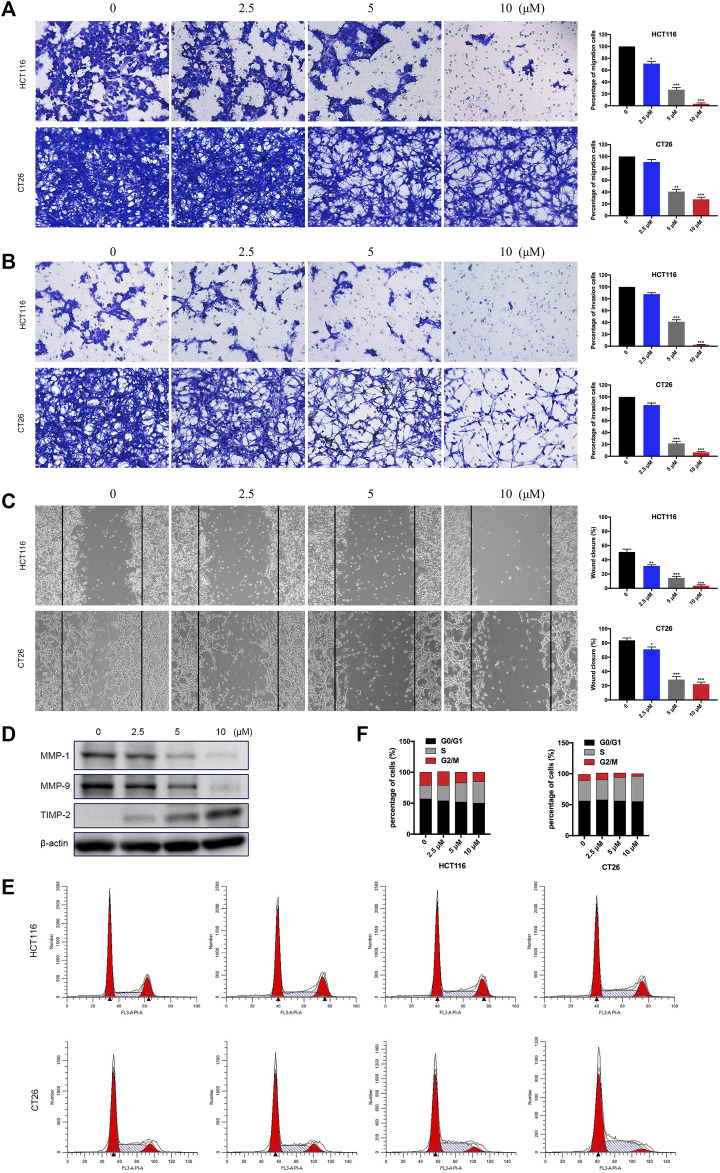
BTD inhibited the migration and invasion ability of cells. **(A,B)** Cells were treated with BTD (0, 2.5, 5, or 10 μM) for 24 h and subjected to migration **(A)** and invasion **(B)** assays, and then photographed (×100). **(C)** Cells were seeded, and a “wound” was created, then incubated with BTD (0, 2.5, 5, or 10 μM) for 24 h and photographed (×100). The lines indicate the area occupied by the initial scraping, and migrated cells were quantified. **(D)** The levels of MMP2, MMP9, and TIMP2 were detected using Western blotting. **(E,F)** Flow cytometry was used to analyze the cell cycle distribution. The values are shown as histograms.

The migration and invasion of tumor cells play critical roles in the process of tumor genesis and progress. We performed migration, invasion, and wound healing assays to measure the effect of BTD on the metastatic ability of tumor cells. As displayed in Figures 5A–C, BTD inhibited the migration of CRC cells in a dose-dependent manner, consistent with the results observed in transwell invasion assays. We also conducted Western blot analysis to investigate the expression of proteins that are considered to be related to cell migration and invasion, including MMP2, MMP9, and TIMP2 (Deryugina and Quigley, 2015). As shown in Figure 5D, BTD decreased the expression of metastasis promoting proteins—MMP2 and MMP9 and increased the expression of metastasis suppressor—TIMP2. To confirm whether the negative effect of BTD on cell viability contributed to cell cycle arrest, we used FCM to assess the cell cycle distribution. Figures 5E, F showed that BTD slightly induced S phase arrest, although the difference was not statistically significant. Collectively, these data indicated that BTD suppressed the migration and invasion ability of CRC cells.

### BTD suppressed tumor growth *in vivo*

**FIGURE 6:**
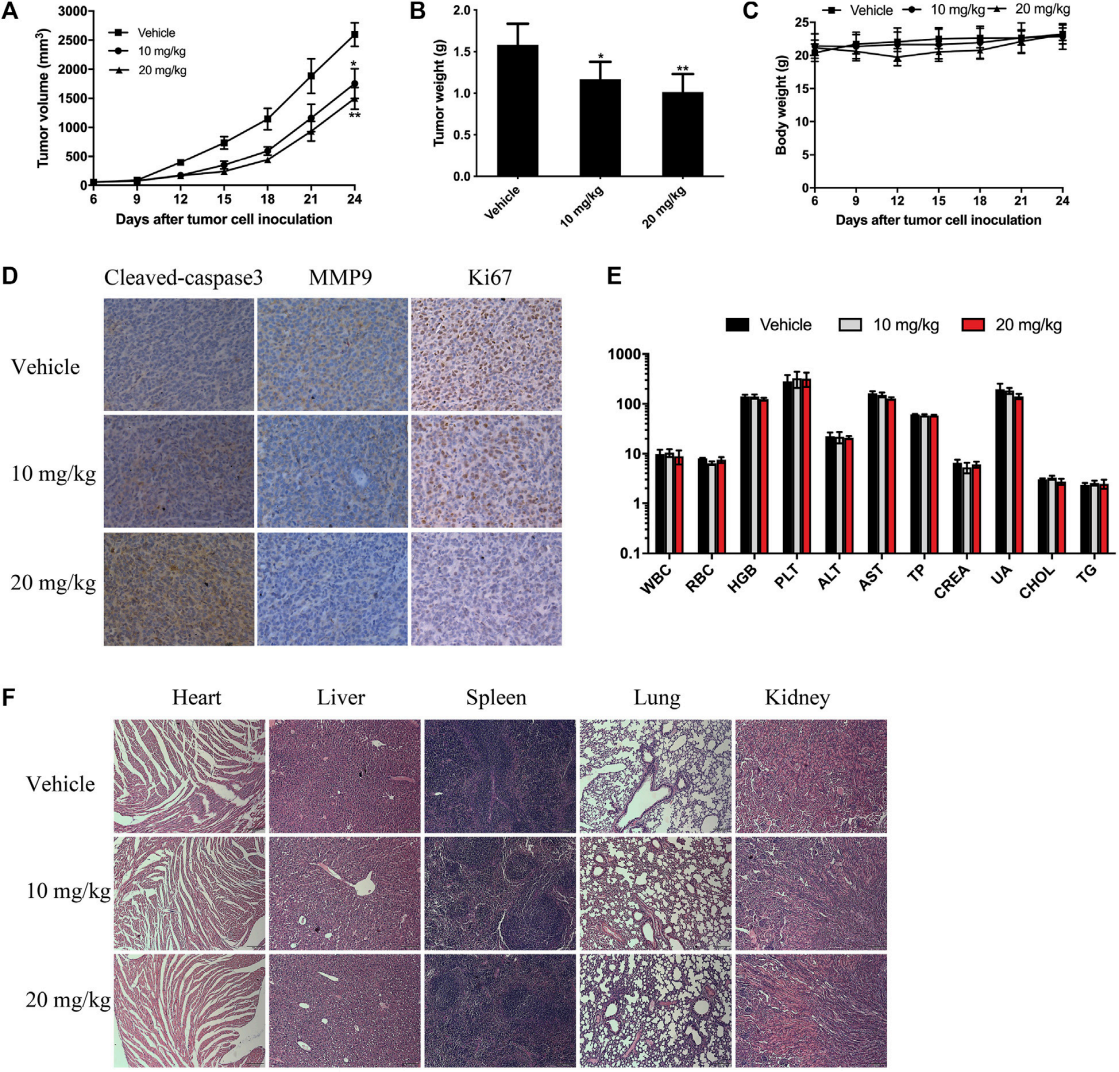
BTD suppressed tumor growth *in vivo*. CT26 tumor-bearing mice were treated with 10 mg/kg or 20 mg/kg of BTD, or vehicle. **(A)** Tumor volume curve of the mice. **(B)** The bars represent tumor weight. **(C)** Body weight curve of the mice. **(D)** IHC staining for cleaved caspase-3, MMP9, and Ki-67 in tumor tissues. **(E)** Serological and hematological analyses. The units of the parameters are as follows: white blood cell (WBC) red blood cell (RBC) (10^12^/L), hemoglobin (HGB) (g/L), platelet (PLT) (10^9^/L), alanine aminotransferase (ALT), aspartate aminotransferase (AST) (U/L), total protein (TP) (g/L), creatinine (CREA), uric acid (UA) (μM), cholesterol (CHOL) (mM), and triglyceride (TG). **(F)** H&E staining of the heart, liver, spleen, lung, and kidney (×100).

To further evaluate the antitumor effect of BTD *in vivo*, we treated CT26 tumor-bearing mice with BTD daily at doses of 10 or 20 mg/kg, respectively. As shown in Figures 6A, B, BTD treatment leaded to retarded tumor progression and significantly lower tumor weight compared with the vehicle group. However, there was no significant difference in body weight between the two groups of mice (Figure 6C). The volumes of the tumors were suppressed by 32.5% and 42.3% in the 10 mg/kg group and 20 mg/kg group, respectively, compared with the vehicle group. We stained cleaved caspase-3, MMP9, and Ki-67 by IHC to detect the effects of BTD on apoptosis and ability to metastasize. After BTD treatment, expression of MMP9 and Ki-67 was downregulated, whereas expression of cleaved caspase-3 was upregulated (Figure 6D). There was no pathological change observed in the hematological and serological examination, compared with the control group (Figure 6E). H&E staining was performed in the major organs, including the heart, liver, spleen, lung, and kidney, and no pathological changes were observed (Figure 6F). To summarize, these results suggested that BTD exhibited strong antitumor activity and was relatively safe.

## Discussion

Apoptosis is the result of an ordered cascade of enzymatic events, which maintain the stability of the internal environment. However, this process is deregulated in various cancers, making cancer cells difficult to kill. Drugs that could restore the normal apoptotic pathways may be promising candidates as tumor therapies (Wong, 2011; Mohammad et al., 2015; Carneiro and El-Deiry, 2020). Generally, apoptosis is induced by two pathways: the mitochondrial-mediated intrinsic pathway, and the cell death receptor-mediated extrinsic pathway. Mitochondria are the main sites at which cells produce ROS. Excessive accumulation of ROS destroys the integrity of mitochondrial membrane, causing the opening of mitochondrial membrane transport pores, reducing the mitochondrial transmembrane potential, and leading to mitochondrial pathway-mediated apoptosis (Zorov et al., 2014; Burke, 2017). In this study, we found that BTD promoted ROS accumulation in tumor cells, thus leading to the loss of mitochondrial transmembrane potential, and ultimately inducing apoptosis of tumor cells. All these suggested that BTD-induced apoptosis in tumor cells via the ROS-mediated mitochondrial apoptosis pathway.

Bcl-2 family proteins play a key role in the induction of intrinsic apoptosis. These proteins include the anti-apoptotic proteins Bcl-2 and Bcl-x and pro-apoptotic proteins Bax and Bad (Singh et al., 2019; Warren et al., 2019). Several antitumor agents have been reported to exert antitumor effects via the intrinsic pathway. This investigation demonstrated that BTD resulted in the loss of mitochondrial transmembrane potential, downregulated the expression of Bcl-2 and Bcl-x, and increased the expression of Bax, Bim, and Bad, and increased caspase-3 cleavage. BTD could also lead to translocation of Bax from the cytoplasm to the mitochondria, and promote release of Cyto C from the mitochondria into the cytoplasm. Therefore, the mitochondria-mediated intrinsic apoptotic pathway might, at least partly, contribute to the eventual apoptosis of CRC cells induced by BTD.

The migration and invasion of tumor cells is fundamental to tumorigenesis. To metastasize from the primary focus, tumor cells need to remodel the extracellular matrix (ECM) and disrupt tissue barriers that usually prevent them from entering blood vessels, lymphatic vessels, or body cavities. MMPs can degrade the ECM and facilitate the entry of tumor cells into the vascular system (Friedl and Wolf, 2003; Novikov et al., 2021). Tissue inhibitor of metalloproteinase (TIMP) can inhibit the expression of MMPs. The balance between them plays a key role in maintaining the integrity of the cell basement membrane. It has been reported that MMP2, MMP9, and TIMP2 are involved in CRC metastasis (Nagase et al., 2006). Our study demonstrated that BTD suppressed the migration and invasion ability of tumor cells *in vitro*. Western blot analysis exhibited that BTD downregulated the expression of MMP2 and MMP9 and upregulated the expression of TIMP2, which also partially supported its anti-metastasis effect.

In our study, BTD was found to significantly inhibit tumorigenesis *in vivo*, and few adverse effects were observed in the experimental animals. IHC staining of tumor tissues demonstrated increased expression level of cleaved caspase-3 and decreased expression levels of MMP9 and Ki-67, which is consistent with the results observed *in vitro*, indicating that BTD could inhibit tumor cell proliferation and metastasis, and induce apoptosis of tumor cells *in vivo*.

## Conclusion

To summarize, this novel BTD could inhibit the proliferation, migration, and invasion of CRC cells, and induce apoptosis through the ROS-mediated mitochondrial intrinsic pathway. The compound exhibited impressive antitumor effect without apparent toxicity in a mouse model. Although this is the first research to identify antitumor activity of BTD in CRC, all the above evidences are sufficiently compelling to support that BTD may be a promising candidate treatment for CRC.

## Data Availability

The datasets presented in this study can be found in online repositories. The names of the repository/repositories and accession number(s) can be found in the article/supplementary material.

## Abbreviations

CRC, Colorectal cancer; BTD, benzothiazole derivative; ^1^H-NMR, hydrogen-1 nuclear magnetic resonance; DMSO, dimethyl sulfoxide; MTT, 3-(4, 5-dimethylthiazol-2-yl)-2, 5-diphenyltetrazoliumbromide; Rh123, 2-(6-Amino-3-imino-3H-xanthen-9-yl) benzoic acid methyl ester; DCFH-DA, 20,70-dichlorodihydrofluorescein diacetate; EdU, 5-ethynyl-2-deoxyuridine; RPMI-1640, Roswell Park Memorial Institute-1640; DMEM, Dulbecco’s modified Eagle’s medium; FBS, fetal bovine serum; GSEA, gene set enrichment analysis; PI, propidium iodide; FCM, flow cytometry; SDS-PAGE, sodium dodecyl sulfate-polyacrylamide gel; PVDF, polyvinylidene difluoride; SPF, specific-pathogen-free; Tis, tumor inhibition rates; H&E, hematoxylin-eosin; IHC, immunohistochemical; ROS, reactive oxygen species; Cyto C, Cytochrome C; ECM, extracellular matrix; MMPs, matrix metalloproteinases; TIMP, tissue inhibitor of metalloproteinase.

## References

[B1] Ahmed K. Yellamelli Valli Venkata S. Mohammed N. A. Sultana F. Methuku K. R. (2012). Recent advances on structural modifications of benzothiazoles and their conjugate systems as potential chemotherapeutics. Expert Opin. Investig. Drugs 21, 619–635. 10.1517/13543784.2012.676043 22493977

[B2] Bedoui S. Herold M. J. Strasser A. (2020). Emerging connectivity of programmed cell death pathways and its physiological implications. Nat. Rev. Mol. Cell. Biol. 21, 678–695. 10.1038/s41580-020-0270-8 32873928

[B3] Bien J. Lin A. (2021). A review of the diagnosis and treatment of metastatic colorectal cancer. JAMA 325, 2404–2405. 10.1001/jama.2021.6021 34129003

[B4] Biller L. H. Schrag D. (2021). Diagnosis and treatment of metastatic colorectal cancer: A review. JAMA 325, 669–685. 10.1001/jama.2021.0106 33591350

[B5] Burke P. J. (2017). Mitochondria, bioenergetics and apoptosis in cancer. Trends Cancer 3, 857–870. 10.1016/j.trecan.2017.10.006 29198441 PMC5957506

[B6] Carneiro B. A. El-Deiry W. S. (2020). Targeting apoptosis in cancer therapy. Nat. Rev. Clin. Oncol. 17, 395–417. 10.1038/s41571-020-0341-y 32203277 PMC8211386

[B7] Deryugina E. I. Quigley J. P. (2015). Tumor angiogenesis: MMP-mediated induction of intravasation- and metastasis-sustaining neovasculature. Matrix Biol. 44-46, 94–112. 10.1016/j.matbio.2015.04.004 25912949 PMC5079283

[B8] Fesik S. W. (2005). Promoting apoptosis as a strategy for cancer drug discovery. Nat. Rev. Cancer 5, 876–885. 10.1038/nrc1736 16239906

[B9] Friedl P. Wolf K. (2003). Tumour-cell invasion and migration: Diversity and escape mechanisms. Nat. Rev. Cancer 3, 362–374. 10.1038/nrc1075 12724734

[B10] Irfan A. Batool F. Zahra Naqvi S. A. Islam A. Osman S. M. Nocentini A. (2020). Benzothiazole derivatives as anticancer agents. J. Enzyme Inhib. Med. Chem. 35, 265–279. 10.1080/14756366.2019.1698036 31790602 PMC6896476

[B11] Kamal A. Syed M. A. Mohammed S. M. (2015). Therapeutic potential of benzothiazoles: A patent review (2010 - 2014). Expert Opin. Ther. Pat. 25, 335–349. 10.1517/13543776.2014.999764 25579497

[B12] Kuipers E. J. Grady W. M. Lieberman D. Seufferlein T. Sung J. J. Boelens P. G. (2015). Colorectal cancer. Nat. Rev. Dis. Prim. 1, 15065. 10.1038/nrdp.2015.65 27189416 PMC4874655

[B13] Letai A. G. (2008). Diagnosing and exploiting cancer's addiction to blocks in apoptosis. Nat. Rev. Cancer 8, 121–132. 10.1038/nrc2297 18202696

[B14] Mohammad R. M. Muqbil I. Lowe L. Yedjou C. Hsu H. Y. Lin L. T. (2015). Broad targeting of resistance to apoptosis in cancer. Semin. Cancer Biol. 35, S78–S103. 10.1016/j.semcancer.2015.03.001 25936818 PMC4720504

[B15] Nagase H. Visse R. Murphy G. (2006). Structure and function of matrix metalloproteinases and TIMPs. Cardiovasc Res. 69, 562–573. 10.1016/j.cardiores.2005.12.002 16405877

[B16] Novikov N. M. Zolotaryova S. Y. Gautreau A. M. Denisov E. V. (2021). Mutational drivers of cancer cell migration and invasion. Br. J. Cancer 124, 102–114. 10.1038/s41416-020-01149-0 33204027 PMC7784720

[B17] Omar A. M. E. Aboulwafa O. M. Amr M. E. El-Shoukrofy M. S. (2021). Antiproliferative activity, enzymatic inhibition and apoptosis-promoting effects of benzoxazole-based hybrids on human breast cancer cells. Bioorg Chem. 109, 104752. 10.1016/j.bioorg.2021.104752 33657444

[B18] Saifutiarova A. E. Fedorov Y. V. Tsvetkov V. B. Rustamova D. A. Gulakova E. N. Chmelyuk N. S. (2021). Photochemical synthesis, intercalation with DNA and antitumor evaluation *in vitro* of benzo[d]thiazolo[3,2-a]quinolin-10-ium derivatives. Bioorg Chem. 115, 105267. 10.1016/j.bioorg.2021.105267 34426158

[B19] Simard J. Kamath S. Kircher S. (2019). Survivorship guidance for patients with colorectal cancer. Curr. Treat. Options Oncol. 20, 38. 10.1007/s11864-019-0635-4 30937550

[B20] Singh R. Letai A. Sarosiek K. (2019). Regulation of apoptosis in health and disease: The balancing act of BCL-2 family proteins. Nat. Rev. Mol. Cell. Biol. 20, 175–193. 10.1038/s41580-018-0089-8 30655609 PMC7325303

[B21] Srinivas U. S. Tan B. W. Q. Vellayappan B. A. Jeyasekharan A. D. (2019). ROS and the DNA damage response in cancer. Redox Biol. 25, 101084. 10.1016/j.redox.2018.101084 30612957 PMC6859528

[B22] Sung H. Ferlay J. Siegel R. L. Laversanne M. Soerjomataram I. Jemal A. (2021). Global cancer statistics 2020: GLOBOCAN estimates of incidence and mortality worldwide for 36 cancers in 185 countries. CA Cancer J. Clin. 71, 209–249. 10.3322/caac.21660 33538338

[B23] Wang Z. Shi X. H. Wang J. Zhou T. Xu Y. Z. Huang T. T. (2011). Synthesis, structure-activity relationships and preliminary antitumor evaluation of benzothiazole-2-thiol derivatives as novel apoptosis inducers. Bioorg Med. Chem. Lett. 21, 1097–1101. 10.1016/j.bmcl.2010.12.124 21262571

[B24] Warren C. F. A. Wong-Brown M. W. Bowden N. A. (2019). BCL-2 family isoforms in apoptosis and cancer. Cell. Death Dis. 10, 177. 10.1038/s41419-019-1407-6 30792387 PMC6384907

[B25] Wong R. S. (2011). Apoptosis in cancer: From pathogenesis to treatment. J. Exp. Clin. Cancer Res. 30, 87. 10.1186/1756-9966-30-87 21943236 PMC3197541

[B26] Zhilitskaya L. V. Shainyan B. A. Yarosh N. O. (2021). Modern approaches to the synthesis and transformations of practically valuable benzothiazole derivatives. Molecules 26, 2190. 10.3390/molecules26082190 33920281 PMC8070523

[B27] Zorov D. B. Juhaszova M. Sollott S. J. (2014). Mitochondrial reactive oxygen species (ROS) and ROS-induced ROS release. Physiol. Rev. 94, 909–950. 10.1152/physrev.00026.2013 24987008 PMC4101632

